# Supplementary material for: Iron Supplementation Eliminates Antagonistic Interactions Between Root-Associated Bacteria
Source: Front Microbiol. 2020 Jul 22;11:1742. doi: 10.3389/fmicb.2020.01742 (PMC7387576; doi:10.3389/fmicb.2020.01742)

Supplemental Figure 1

Actinobacteria:
Arthrobacter sp.
Leifsonia sp.

Bacteroidetes:
Chryseobacterium sp.
Flavobacterium sp.

Firmicutes:
Bacillus flexus
Paenibacillus sp.

α-Proteobacteria:
Agrobacterium rhizogenes
Brevundimonas sp.

β-Proteobacteria:
Ralstonia sp.

γ-Proteobacteria:
Acinetobacter sp. 01
Acinetobacter sp. 02

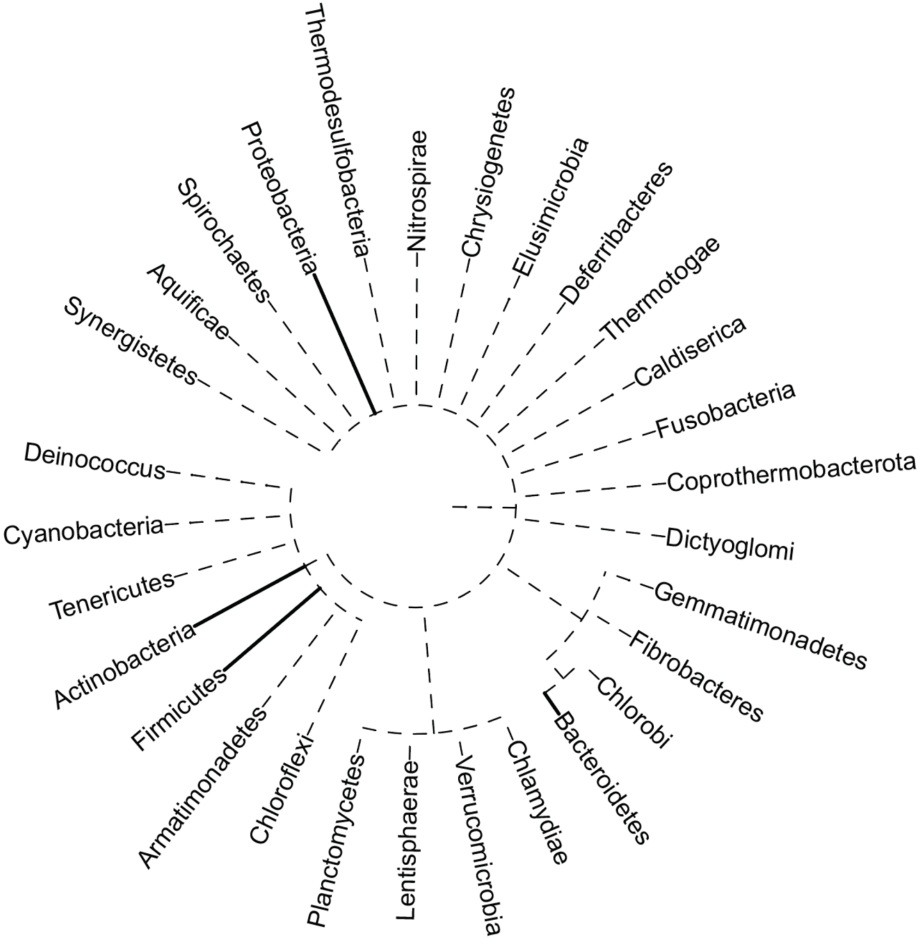

Supplemental Figure 2

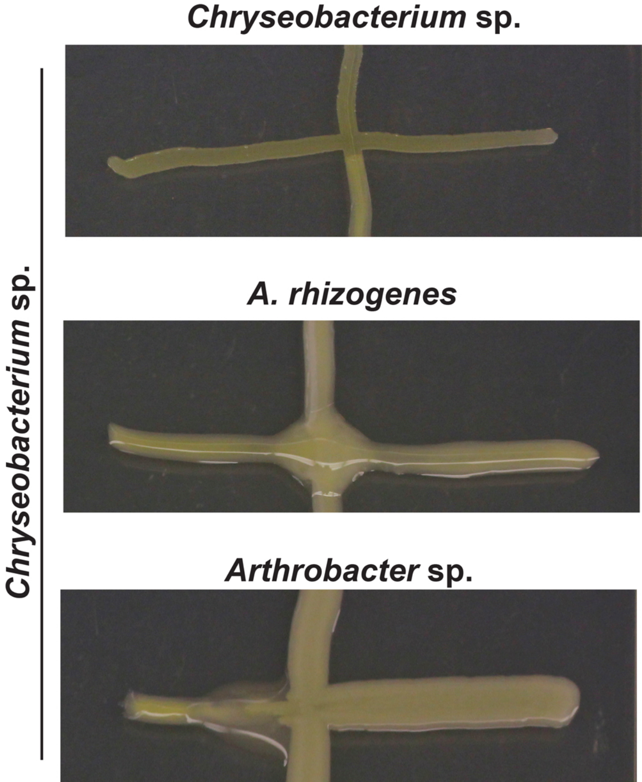

Supplemental Figure 3

A. rhizogenes **Chryseobacterium** **sp.**

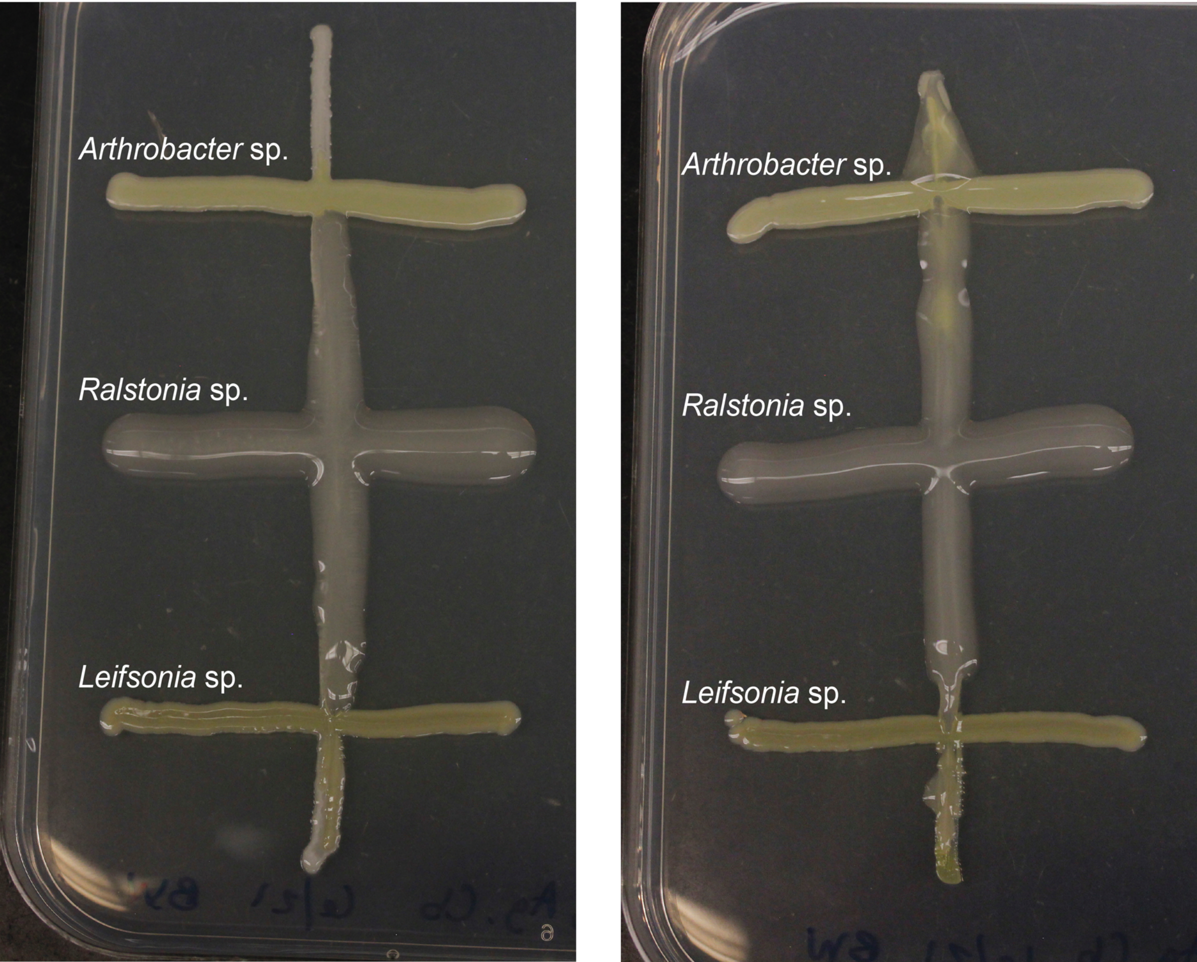

Supplemental Figure 4

P. putida KT2440 grown in:

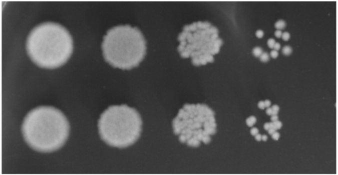

Supplemental Figure 5

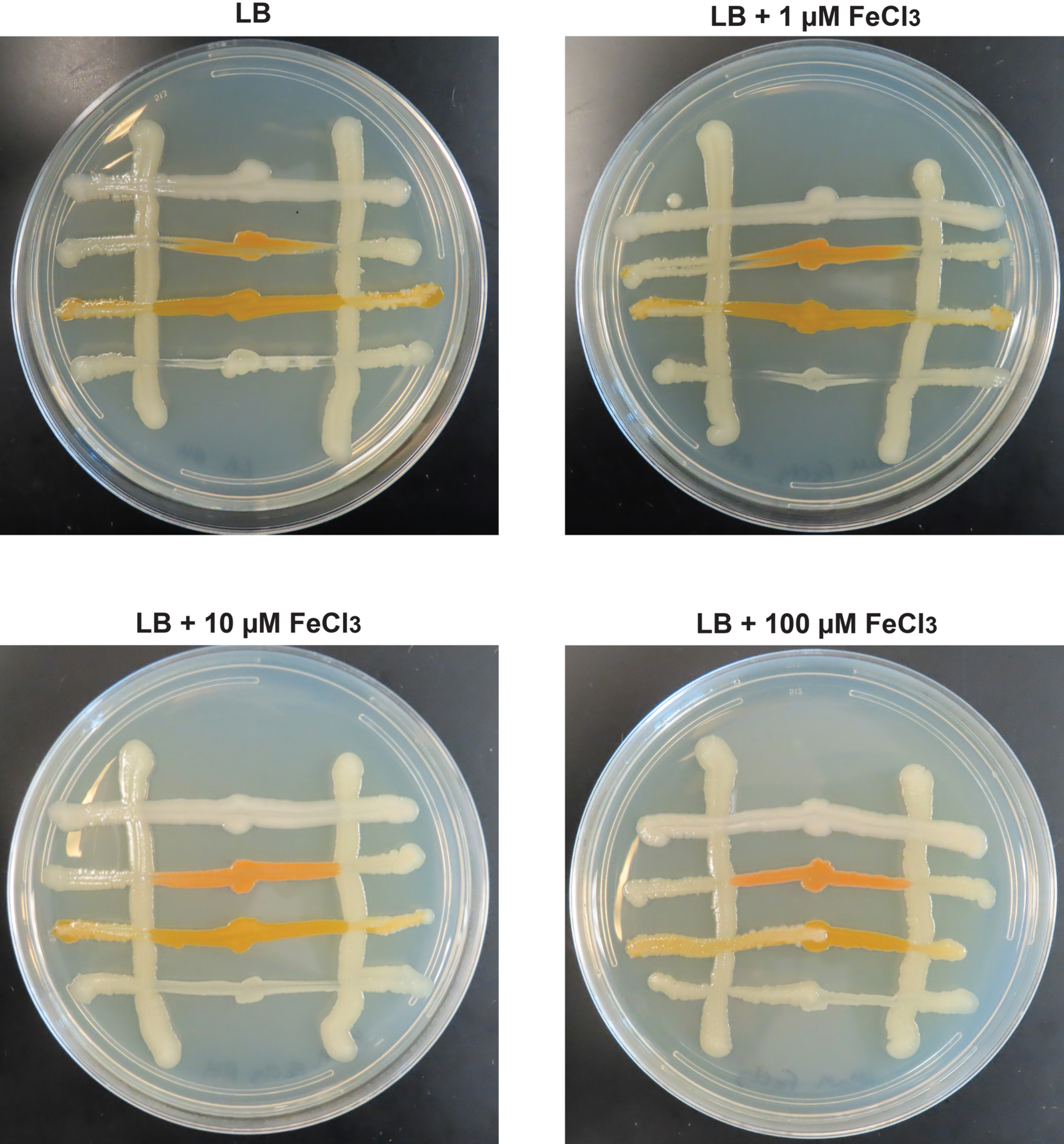

Supplement: FIGURE S1 — Phylogeny of rhizobacteria used in this study. Genus-level description of selected rhizobacteria and their representation within major bacterial phyla. The bold, solid lines in the phylogenetic tree indicate lineages represented by the isolates used in this study. The phylogenetic tree was generated using NCBI taxonomy IDs and visualized using iTOL V4. [file Data_Sheet_2.pdf]
